# *In Situ* Staining and Laser Capture Microdissection of Lymph Node Residing SIV Gag-Specific CD8^+^ T cells—A Tool to Interrogate a Functional Immune Response *Ex Vivo*

**DOI:** 10.1371/journal.pone.0149907

**Published:** 2016-03-17

**Authors:** Annelie Tjernlund, Adam Burgener, Jessica M. Lindvall, Tao Peng, Jia Zhu, Lars Öhrmalm, Louis J. Picker, Kristina Broliden, M. Juliana McElrath, Lawrence Corey

**Affiliations:** 1 Department of Medicine Solna, Unit of Infectious Diseases, Center for Molecular Medicine, Karolinska Institutet, Karolinska University Hospital, L8:01, 17176 Stockholm, Sweden; 2 National Laboratory for HIV Immunology, Public Health Agency of Canada, Winnipeg, Manitoba, Canada; 3 Department of Medical Microbiology, University of Manitoba, 730 William Ave. Winnipeg, MB, Canada; 4 Department of Biosciences and Nutrition, Karolinska Institutet, Karolinska University Hospital Huddinge, Huddinge, Stockholm, Sweden; 5 Vaccine and Infectious Disease Division, Fred Hutchinson Cancer Research Center, Seattle, WA, United States of America; 6 Department of Medicine, University of Washington, Seattle, WA, United States of America; 7 Department of Laboratory Medicine, University of Washington, Seattle, WA, United States of America; 8 Department of Pathology, Vaccine and Gene Therapy Institute, and the Oregon National Primate Research Center, Oregon Health & Science University, Beaverton, OR, United States of America; University of Pittsburgh, UNITED STATES

## Abstract

While a plethora of data describes the essential role of systemic CD8^***+***^ T cells in the control of SIV replication little is known about the local *in situ* CD8^***+***^ T cell immune responses against SIV at the intact tissue level, due to technical limitations. *In situ* staining, using GagCM9 Qdot 655 multimers, were here combined with laser capture microdissection to detect and collect SIV Gag CM9 specific CD8^+^ T cells in lymph node tissue from SIV infected rhesus macaques. CD8^+^ T cells from SIV infected and uninfected rhesus macaques were also collected and compared to the SIV GagCM9 specific CD8^+^ T cells. Illumina bead array and transcriptional analyses were used to assess the transcriptional profiles and the three different CD8^+^ T cell populations displayed unique transcriptional patterns. This pilot study demonstrates that rapid and specific immunostaining combined with laser capture microdissection in concert with transcriptional profiling may be used to elucidate phenotypic differences between CD8^+^ T cells in SIV infection. Such technologies may be useful to determine differences in functional activities of HIV/SIV specific T cells.

## Introduction

Replication of human immunodeficiency virus (HIV) and simian immunodeficiency virus (SIV), the simian equivalent of HIV, is highly variable, as is the host immune response to the infection. Differences in host genetics and adaptive immunity influence the clinical course and progression of the infection. However, CD8^+^ T cells, in particular, may play a pivotal role in controlling both HIV and SIV replication [1–11]. For example, slow progression of HIV/SIV infection is associated with the ability to mount a diverse CD8^+^ T cell restricted response (HLA/MHC class I restricted response) [12]. Humans that have an overrepresentation of HLA-B*27, HLA-B*57 or HLA-B*28 alleles and rhesus macaques (RMs) that have an overrepresentation of Mamu-A*01, Mamu-B*08, or Mamu-B*17 alleles are associated with a slow HIV/SIV disease progression [12]. CD8^+^ T cell responses to specific epitopes are associated with slower progression rates, but not all HIV/SIV specific CD8^+^ T cells are uniformly capable of preventing HIV/SIV replication [2, 13]. GagCM9 is an immunodominant cytotoxic CD8^+^ T cell epitope, which is restricted by the Mamu A*01 allele, and is well characterized in non-human primate (NHP) models, both in SIV infection and SIV vaccine models [12, 14–16]. GagCM9 specific CD8^+^ T cells are suggested to have multifunctional capacity (e.g. degranulate and produce several cytokines at the same time) as well as having access to lymphoid tissues where the primary sites of viral replication occur [17, 18].

While a plethora of data describes the essential role of systemic CD8^***+***^ T cells in the control of HIV/SIV replication, little is known about the local *in situ* CD8^***+***^ T cell immune responses against HIV/SIV at the intact tissue level [17–19]. Since the distribution and function of immune cells naturally differ between blood, secretions and tissue sites, it is important to study the immune response in these compartments. Furthermore, since HIV/SIV predominantly replicate in lymphoid tissue it is of major importance to study the *in situ* immune response, including the CD8^+^ T cell response, against HIV/SIV directly in these tissues [20–26]. We have previously shown that it is possible to detect GagCM9 specific CD8^+^ T cells in cryopreserved lymphoid tissue from chronically SIV infected RMs with the use of GagCM9 Qdot 655 multimers (Qdot 655 conjugated with the Mamu-A*01 MHC Class I allele loaded with the SIVmac239 peptide Gag_181-189_CM9; Gag CM9) [27]. This report describes a pilot study to evaluate the use of these Gag CM9 Qdot 655 multimers for *in situ* staining followed by laser capture microdissection (LCM) and the subsequent gene transcriptional profiles of these cell populations.

## Materials and Methods

### Animals, Specimen collection and Ethical statement

Submandibular and mesenteric lymph nodes biopsies were obtained from four purpose-bred RMs (*Macaca mulatta*) of Indian genetic background; three of the RMs were chronically SIVmac239 infected Mamu-A*01 positive RMs (animal A, B and C) and one RM were Mamu-A*01 negative, SIV uninfected RM (animal D). SIVmac239 infections were initiated with intravenous injection of 5 ng equivalents of SIV p27. The lymph nodes biopsies used in this study were obtained at necropsy (77–85 days post infection), collected by clinical veterinarians. The plasma viral load and the SIV RNA content within the lymph nodes of the three SIV infected RMs, at time of necropsy has been described previously [27]. Shortly, plasma viral load for animal A was 3.3 x 10^6^, for animal B it was <30, and for animal C it was 50 SIV RNA copy/ml plasma. The SIV RNA content in the submandibular lymph nodes for animal A was 29, for animal B it was 28, and for animal C it was120 RNA copies/250 ng total RNA. The SIV RNA content in the mesenteric lymph nodes for animal A was 9 736, for animal B it was 324, and for animal C it was 7 RNA copies/250 ng total RNA. The biopsies were snap frozen in OCT media (Sakura Finetek USA Inc. Torrance, CA) and kept at -80°C until sectioning.

Specimens from animal A, B and C were obtained from the Oregon National Primate Research Center (Beaverton, OR) (IACUC ID: 0569 and 0631). Specimens from animal D were obtained from the Washington National Primate Research Center at the University of Washington (Seattle, WA), NIH grant RR00166 and from the National Center for Research Resources and the Office of Research Infrastructure Programs (ORIP) of the National Institutes of Health through Grant Number P51 OD 010425 (IACUC ID: 4140–01; Tissue Distribution Program). The study protocols were approved by the Oregon National Primate Research Center’s and the University of Washington Institutional Animal Care and Use Committee (with membership constituted to comply with NIH policy and Animal Welfare Act regulations) under the NIH Office for Laboratory Animal Welfare. The RMs were housed and cared in accordance with standards of the US National Institutes of Health Guide for the Care and Use of Laboratory Animals [28]. The RMs were housed in indoor or indoor-outdoor facilities with twice-daily collection of waste pans, in cages that were sanitized in a central cage washer at least every 2 weeks. They were fed with commercially prepared primate feed milled, supplemented daily with fruits. Fresh potable water was provided by the municipal water district via automatic water systems. Environmental enrichments were provided (toys and a variety of complex foraging devices). The macaques were observed for species-specific behaviors, food and water consumption, and urine and feces production for reporting of abnormalities to the attending veterinarian. All animals were evaluated for clinical signs of disease on a daily basis and a clinical veterinarian was responsible for determining if an animal was experiencing any pain or suffering. The animals were euthanized humanely by an overdose of anesthetic (e.g. >50 mg/kg sodium pentobarbital and exsanguinated via the distal aorta) after completion of the experiments.

### Synthesis of Gag CM9-Qdot 655 multimers

Qdot 655-conjugated peptide–MHC multimers were formed *in vitro* as previously described [27, 29]. Briefly, biotinylated Mamu-A*01/β_2_m/peptide monomers were produced with the known Mamu-A*01-restricted SIVmac239 peptide Gag_181-189_CM9 (CTPYDINQM: Gag CM9) [14]. Streptavidin-coated Qdot 655 (Life Technologies/Invitrogen, Eugene, OR) were conjugated with a saturating amount of biotinylated Mamu-A*01/β_2_m/peptide monomers.

### Laser capture microdissection of individual cells followed by RNA extraction, cDNA amplification and hybridization to Illumina bead arrays

A rapid immunofluorescent staining method was used to detect SIVGag CM9 specific CD8^+^ cells in lymph node tissue from chronically SIV infected RMs (GagCM9^+^cells_SIV+RMs_), as well as CD8^+^ cells from chronically SIV infected RMs (CD8^+^ cells_SIV+RMs_) and CD8^+^ cells from uninfected RMs (CD8^+^cells_SIV-RMs_). The method was modified from a previous published protocol [27], by using a five-times higher concentration of the GagCM9 Qdot 655 multimer and a ten-times higher concentration of the anti-CD8 antibody. Thereby shorter staining procedure could be achieved to minimize/reduce any potential interaction of RNase activity while preserving the staining intensity. Eight-μm thick sections of the lymph nodes biopsies were mounted on polyethylene naphthalate membrane slide (Carl Zeiss, Thornwood, NY) followed by the addition of the GagCM9-Qdot 655 multimers or Alexa-647 conjugated anti-CD8 antibody (RPA-T8, BD Biosciences, San Diego, CA) for 5 min. The sections were thereafter washed in ice-cold PBS, fixed and dried with ethanol (75%, 95% 100%) and Xylene. The sections were also kept on ice throughout the whole staining procedure to avoid RNA degradation and the staining was performed during less than 15 min. The GagCM9^+^cells_SIV+RMs_, CD8^+^cells_SIV+RMs_ and CD8^+^cells_SIV-RMs_ were visualized and laser microdissected with the use of the Zeiss PALM Microbeam instrument (Carl Zeiss) as previously described [30, 31]. Shortly, a microbeam was used to cut and catapult approximately 100 individual cells, of each specificity, to the designated tube located above the tissue section. Thereafter RNA was extracted from the pooled captured GagCM9^+^cells_SIV+RMs_, CD8^+^ cells_SIV+RMs_ and CD8^+^cells_SIV-RM,_ respectively followed by cDNA amplification and hybridization to Illumina bead arrays as previously described [30, 31]. Briefly, RNA was extracted from the captured cells using Arcturus PicoPure RNA Isolation Kits (Life Technologies/Invitrogen) and quantified with the use of a NanoDrop 1000 Spectometer. One nanogram of total RNA was converted to cDNA and amplified through whole-transcriptome amplification using WT-Ovation Pico RNA Amplification System (NuGEN, San Carlos, CA). Thereafter the cDNA was biotinylated according to the NuGEN protocol and 750 ng of labeled cDNA was hybridized to Illumina Human Ref8 v3 bead arrays in the Shared Resources at Fred Hutchinson Cancer Research Center in Seattle, WA.

### Quantitative PCR

TaqMan quantitative real time PCR (qRT-PCR) assay was performed as previously described [30]. Primer and probe sets (Applied Biosystems, Foster City, CA) were used to detect, amplify and quantify β-actin and CD8A genes. Each sample was run in duplicate and the Ct values for each target gene were normalized to β-actin by using the 2^-dCT^ equation.

### Statistical analysis and hierarchical clustering of microarray data

Illumina Ref8 v3 bead-arrays were processed using Illumina GenomeStudio software and quantile normalized according to the software praxis. Data was then exported and further processed using third party software’s. Probes without an EntrezID were removed, providing 24,487 probes for further analysis. Gene expression was log_2_ transformed to the mean expression value across all samples. To maximize power to detect statistical differences between GagCM9^+^cells_SIV+RMs_, CD8^+^cells_SIV+RMs_ and CD8^+^cells_SIV-RMs_ the log-normalized data from the two different lymph nodes (submandibular and mesenteric) were averaged for differential expression analysis. The mean expression value of each cell population (GagCM9^+^cells_SIV+RMs_, CD8^+^cells_SIV+RMs_ and CD8^+^cells_SIV-RMs_), within each animal (A, B, C and D), was then calculated. Independent sample t-tests (Kruskal-Wallis) were used to detect significant differences between groups. Clustering of genes was generated by unsupervised average linkage hierarchical clustering using Pearson correlation coefficient as the distance metric as described previously [32].

## Results

### Laser capture microdissection of GagCM9^+^cells_SIV+RMs_, CD8^+^cells_SIV+RMs_ and CD8^+^cells_SIV-RMs_

**Fig 1:**
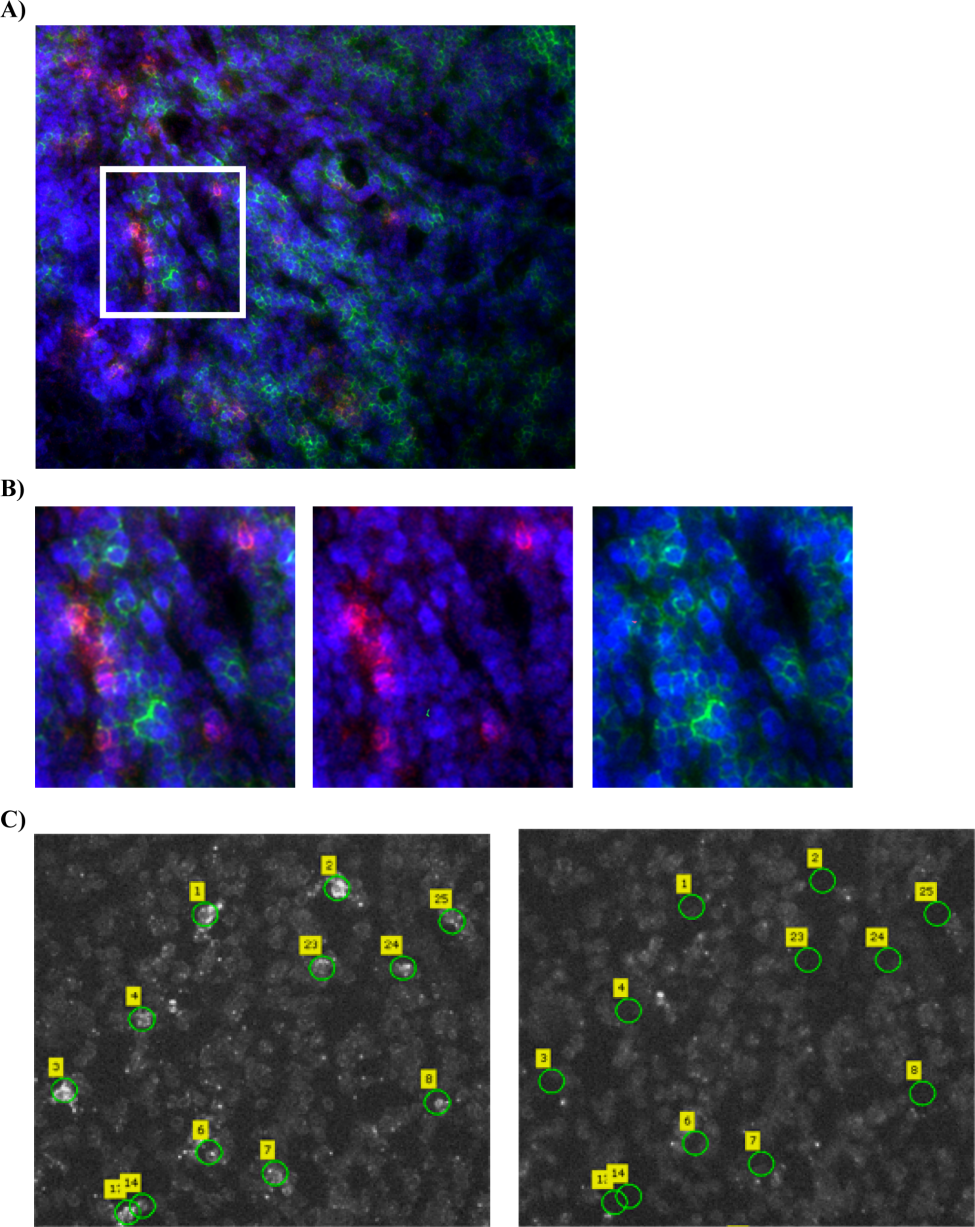
Laser capture microdissection of GagCM9^+^cells_SIV+RMs_, CD8^+^cells_SIV+RMs_ and CD8^+^cells_SIV-RMs_. **A)** Fluorescence images of lymph node tissue sections from an SIV infected RM stained with GagCM9 Qdot 655 multimer (red), CD8 (green) and dapi (blue) showing the abundance of GagCM9^+^ cells. **B)** A magnified view of the region indicated in panel A, demonstrating that the GagCM9^+^cells_SIV+RMs_ cells also express CD8^+^. **C)** Images of lymph node tissue sections from an SIV infected RM stained with CM9 Qdot 655 multimer to detect GagCM9^+^cells_SIV+RMs_. The left images show the tissue section with the selected GagCM9^+^cells_SIV+RMs_ prior to LCM and the images to the right show the same tissue section after the selected cells have been microdissected and collected.

A rapid immunofluorescent staining combined with LCM was used to detect and collect GagCM9^+^cells_SIV+RMs_, CD8^+^ cells_SIV+RMs_ and CD8^+^cells_SIV-RMs_ in cryopreserved tissue sections of lymph nodes obtained from chronically SIV infected and uninfected RMs (Fig 1). We used adjacent tissue sections of lymph node biopsies, which we have previously shown to contain GagCM9 specific CD8^+^ T cells (median 4.5%; range 3.3–6.5% GagCM9^+^cells out of all CD8^+^ T cells) [27], which made it possible to laser-capture microdissect 100 GagCM9^+^cells_SIV+RMs_ per tissue section. RNA was thereafter purified from these laser-captured microdissected cells and converted to cDNA. To confirm that these cells were of CD8 origin we performed qRT-PCR on samples from which we had access to cDNA (3 out of 8 samples of GagCM9^+^cells_SIV+RMs_; 4 out of 5 samples of CD8^+^cells_SIV+RMs_ and 1 out of 2 samples of CD8^+^cells_SIV-RMs_). CD8A, as well asβ-actin (internal control), were detected in all the eight samples assessed (data not shown).

### Gene transcription expression profiles of GagCM9^+^cells_SIV+RMs_ and CD8^+^cells_SIV+RMs_ vs. CD8^+^cells_SIV-RMs_ cells by laser capture microdissection

**Fig 2:**
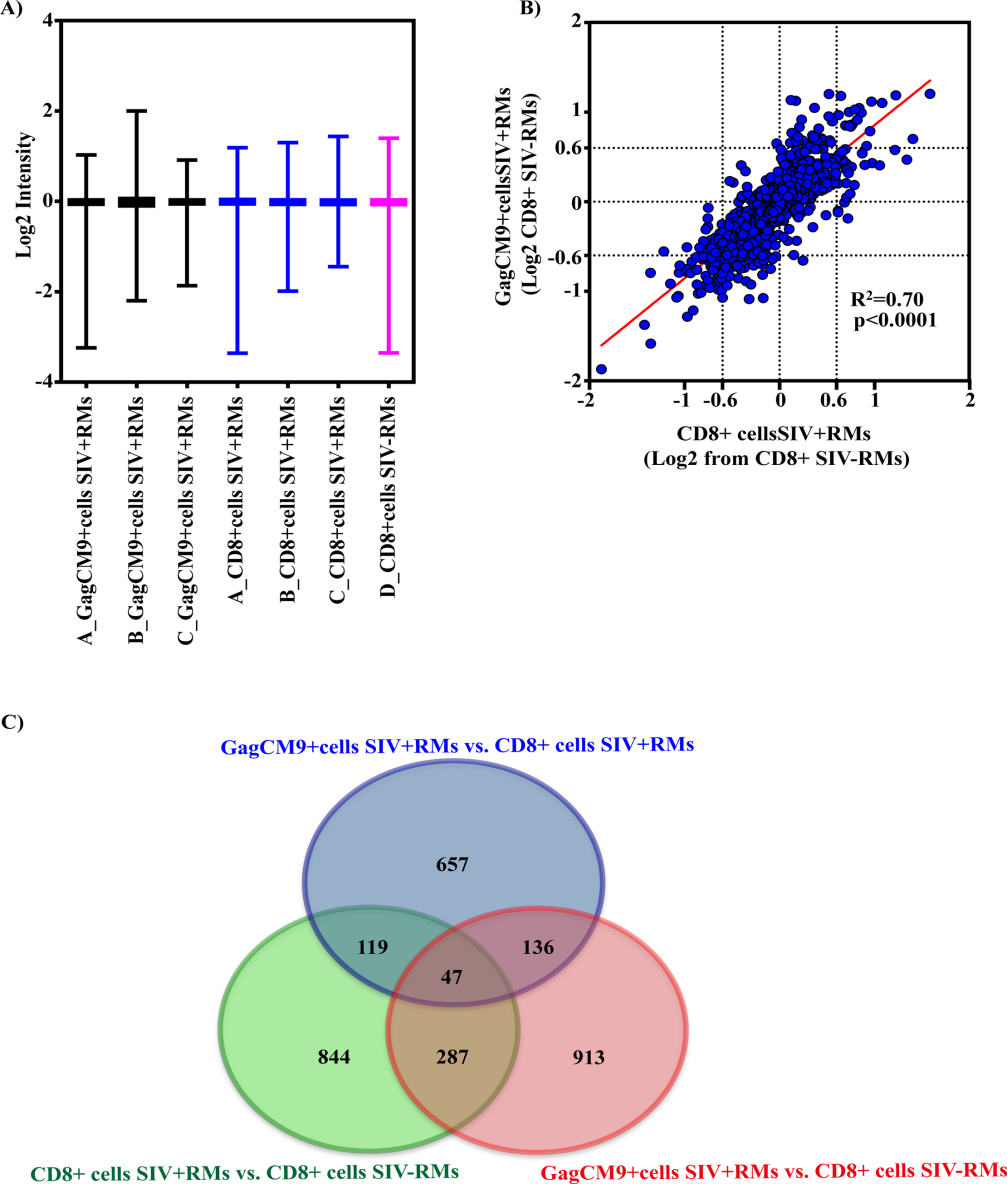
Comparison of gene expression profiles between GagCM9^+^cells_SIV+RMs_ and in CD8^+^cells_SIV+RMs_ vs. CD8^+^cells_SIV-RMs_. **A)** The graph shows the distribution and median of the averaged transformed Log_2_ gene expression showing that the overall gene expression intensity by animal and sample type is similar in all groups. **B)** The graph shows a positive correlation (r^2^ = 0.70) of gene expression differences between GagCM9^+^cells_SIV+RMs_ (Y-axis) and CD8^+^cells_SIV+RMs_ (X-axis) relative to CD8^+^cells_SIV-RMs_. Genes were filtered by those differentially expressed genes found when comparing GagCM9^+^cells_SIV+RMs_ with CD8^+^cells_SIV-RMs_ or CD8^+^cells_SIV+RMs_ with CD8^+^cells_SIV-RMs_ followed by FC calculations. The blue filled circles symbolize the differently expressed genes in at least one comparison between GagCM9^+^cells_SIV+RMs_ and CD8^+^cells_SIV+RMs_ to that of CD8^+^cells_SIV-RMs_ control (p<0.05). **C)** The Venn diagram illustrates how the 2346 genes, which were significantly altered by SIV infection (based on a significance cutoff threshold of p<0.05) overlap between the three comparisons; GagCM9^+^cells_SIV+RMs_ vs. CD8^+^cells_SIV+RMs_, (blue circle); GagCM9^+^cells_SIV+RMs_ vs. CD8^+^cells_SIV-RMs_ (red circle); CD8^+^ cells_SIV+RMs_ vs. CD8^+^cells_SIV-RMs_ (green circle).

The Illumina Human Ref8 v3 bead arrays were used to compare the gene expression profiles between GagCM9^+^cells_SIV+RMs_, CD8^+^cells_SIV+RMs_ and CD8^+^cells_SIV-RMs_. Although underpowered for this purpose with a small animal population size, we reasoned that gene transcriptional profiles may be distinguishable between cell population subsets. Data was quantile normalized (Fig 2A) and genes expression differences compared by t-test between GagCM9^+^cells_SIV+RMs_ with CD8^+^cells_SIV-RMs_ or CD8^+^cells_SIV+RMs_ with CD8^+^cells_SIV-RMs_ followed by fold change (FC) calculations. A positive correlation of gene expression differences was observed (r^2^ = 0.70), indicating a similarity of gene expression changes for the GagCM9^+^cells_SIV+RMs_ and for the CD8^+^cells_SIV+RMs_ relative to the uninfected control (e.g. CD8^+^cells_SIV-RMs_). A relative even distribution of overexpressed and underexpressed genes was observed between GagCM9^+^cells_SIV+RMs_ and the CD8^+^cells_SIV+RMs_ as compared to CD8^+^cells_SIV-RMs_ (Fig 2B). This suggests that GagCM9^+^cells_SIV+RMs_ and CD8^+^cells_SIV+RMs_ located in the lymph nodes of chronically infected RMs show a similar gene expression trend as compared to CD8^+^cells_SIV-RMs_ located in lymph nodes of uninfected RMs.

Using a cutoff threshold of p<0.05, one tenth out of total of 24,487 genes, (2,346 genes; 9.6%) met this significance cutoff threshold when compared to controls (e.g. also differentially expressed to that of CD8^+^cells_SIV-RMs_ control in at least one comparison). This suggests that these genes may be altered due to SIV infection, although as none of these genes met multiple-comparison correction thresholds we cannot discount the possibility of random variation. A Venn diagram illustrates the differences and overlap of the differentially expressed genes (Fig 2C). GagCM9^+^cells_SIV+RMs_ vs. CD8^+^cells_SIV+RMs_ (blue circle) revealed the lowest numbers of differentially expressed genes (959 genes: 491 UP, 468 DOWN) while GagCM9^+^cells_SIV+RMs_ vs. CD8^+^cells_SIV-RMs_ (red circle) reveled the highest numbers of differentially expressed genes (1383 genes: 734 UP, 649 DOWN) followed by the comparison between CD8^+^ cells_SIV+RMs_ vs. CD8^+^cells_SIV-RM_ (green circle) (1297 genes: 652 UP, 645 DOWN). Although not statistically powered, this may indicate that the two CD8^+^ cell populations within the SIV infected RMs are more similar to each other as compared to the CD8^+^ cell populations from SIV infected vs. uninfected RMs. Additionally, 183 genes were shared between the comparison of GagCM9^+^cells_SIV+RMs_ vs. CD8^+^cells_SIV+RMs_ and GagCM9^+^cells_SIV+RMs_ vs. CD8^+^cells_SIV-RMs_; 166 genes were shared between the comparison of GagCM9^+^cells_SIV+RMs_ vs. CD8^+^cells_SIV+RMs_ and CD8^+^ cells_SIV+RMs_ vs. CD8^+^cells_SIV-RMs_; 334 genes were shared between the comparison of GagCM9^+^cells_SIV+RMs_ vs. CD8^+^cells_SIV-RMs_ and CD8^+^ cells_SIV+RMs_ vs. CD8^+^cells_SIV-RMs;_ and 47 genes were shared between all three comparisons.

### Hierarchical clustering analysis distinguishes GagCM9^+^cells_SIV+RMs_ and CD8^+^cells_SIV+RMs_ of infected RMs from CD8^+^cells_SIV-RMs_ of uninfected RM

**Fig 3:**
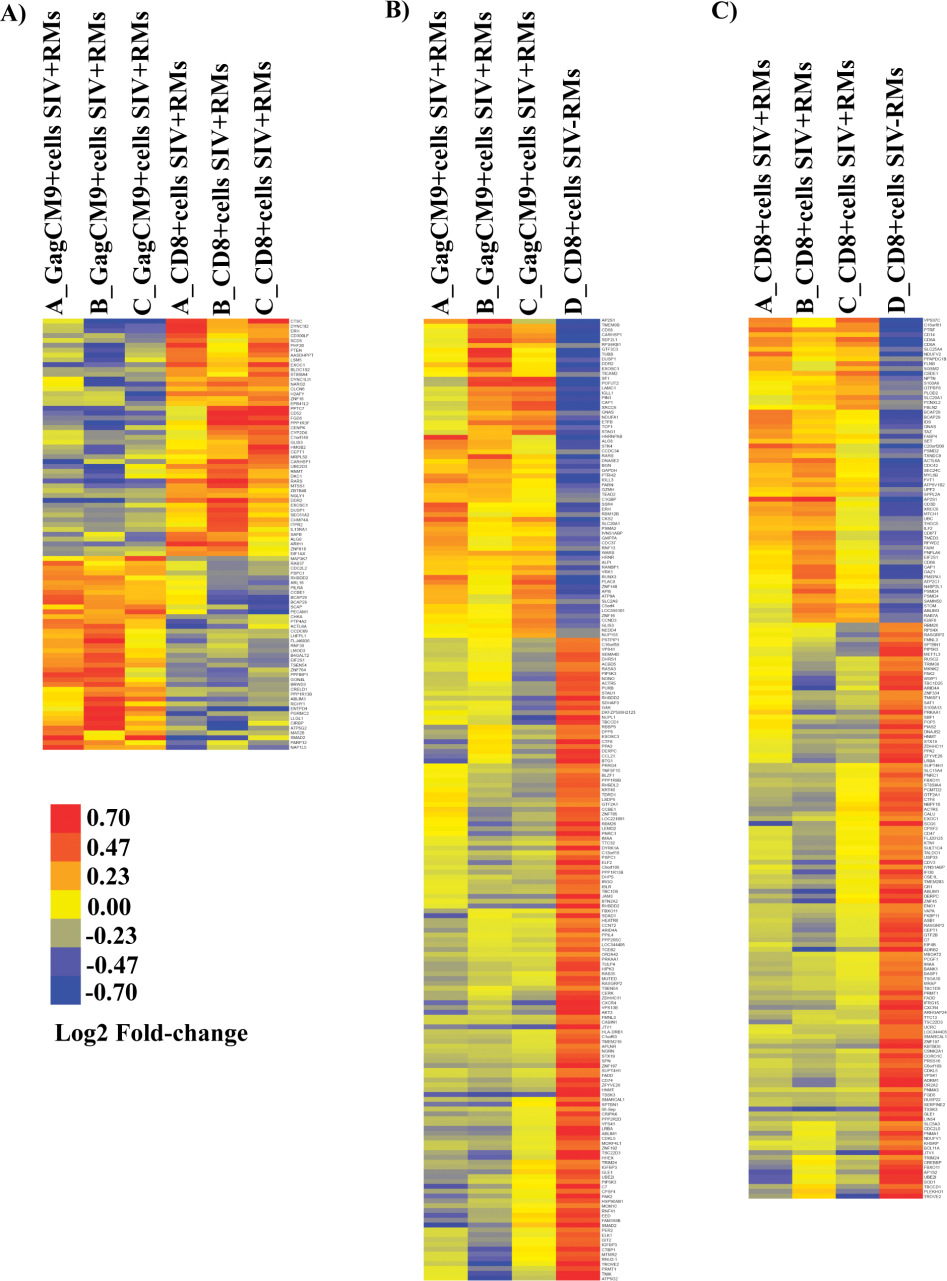
Heat map analysis of GagCM9^+^cells_SIV+RMs_, CD8^+^cells_SIV+RMs_ and CD8^+^cells_SIV-RMs_. The heat map shows discrimination of the gene profiles of **A)** GagCM9^+^cells_SIV+RMs_ vs. CD8^+^cells_SIV+RMs_; **B)** GagCM9^+^cells_SIV+RMs_ vs. CD8^+^cells_SIV-RMs_ and **C)** CD8^+^cells_SIV+RMs_ vs. CD8^+^cells_SIV-RMs_. Independent sample t-tests (Kruskal-Wallis) were used to detect significant differences between groups. Clustering of genes was generated by unsupervised centroid linkage hierarchical clustering using Pearson correlation coefficient as the distance metric. Gene expression levels are shown in color, with red indicating over-abundant expression and blue indicating under-abundant expression.

Hierarchical clustering was performed to better visualize transcriptional profiles of GagCM9^+^cells_SIV+RMs_, CD8^+^cells_SIV+RMs_ and CD8^+^cells_SIV-RMs_. The genes that met a +/- 0.4 log_2_-FC (p<0.05) were used to minimize cluster vertical sizing. This resulted in 84, 200 and 182 differently expressed genes when comparing GagCM9^+^cells_SIV+RMs_ vs. CD8^+^cells_SIV+RMs_ (negative control filtered, e.g. only those genes in context of SIV infection that were significantly different in at least one comparison), GagCM9^+^cells_SIV+RMs_ vs. CD8^+^cells_SIV-RMs_, and CD8^+^cells_SIV+RMs_ vs. CD8^+^cells_SIV-RMs_, respectively (S1–S3 Tables). The heat map of the differentially expressed genes from GagCM9^+^cells_SIV+RMs_ vs. genes from CD8^+^cells_SIV+RMs_ clearly discriminated between these two cell populations, showing both upregulated (51%) and downregulated (49%) gene clusters (Fig 3A). Next, when comparing the differentially expressed genes from GagCM9^+^cells_SIV+RMs_ vs. genes from the CD8^+^cells_SIV-RMs_ 33% of the genes were upregulated and 67% of the genes were downregulated (Fig 3B). A similar pattern was also seen when comparing the differentially expressed genes from CD8^+^cells_SIV+RMs_ as compared to the genes from the CD8^+^cells_SIV-RMs_; 40% of the genes were upregulated and 60% were downregulated (Fig 3C). Furthermore, the transcriptional profile of GagCM9^+^cells_SIV+RMs_ vs. CD8^+^cells_SIV+RMs_ was distinguished from the other two comparisons (GagCM9^+^cells_SIV+RMs_ and CD8^+^cells_SIV+RMs_ vs. CD8^+^cells_SIV-RMs_), which were more comparable to each other. These data suggests that our methods can be utilized to dissect transcriptional profiling of different CD8^+^ cell populations within differing microenvironments.

**Fig 4:**
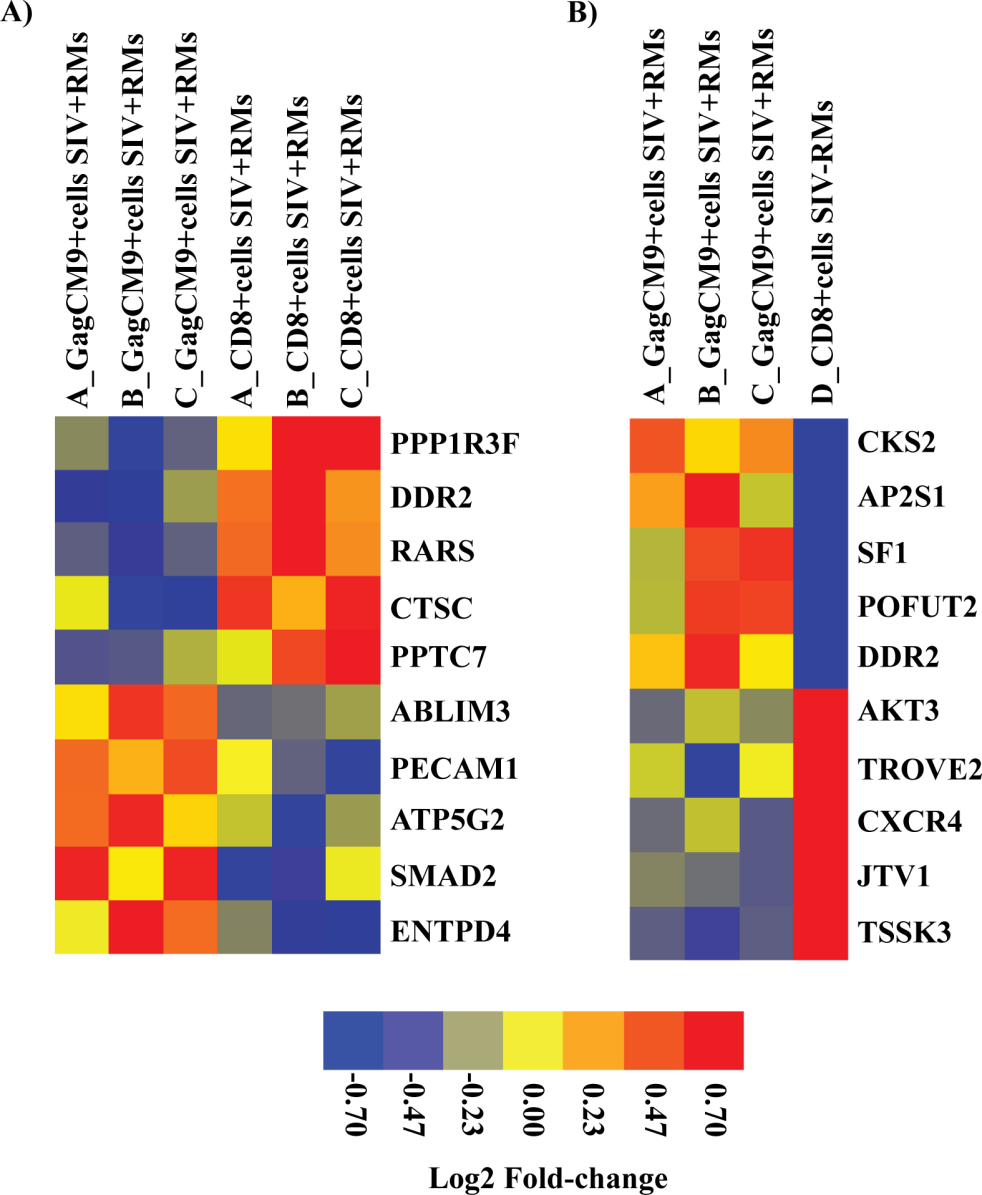
Heat map of top five up and downregulated differentially expressed genes. The heat map shows discrimination of the gene profiles of top five up and downregulated differentially expressed genes in the comparison between **A)** GagCM9^+^cells_SIV+RMs_ vs. CD8^+^cells_SIV+RMs_ and **B)** GagCM9^+^cells_SIV+RMs_ vs. CD8^+^cells_SIV-RMs._ Gene expression levels are shown in color, with red indicating over-abundant expression and blue indicating under-abundant expression.

We classified the top five up- and downregulated differentially expressed genes based on FC value (p<0.05) into functional categories based on gene ontology/annotations (http://www.uniprot.org). Interestingly, one of the five-upregulated genes identified when comparing GagCM9^+^cells_SIV+RMs_ vs. CD8^+^cells_SIV+RMs_ was Cathepsin C (CTSC), a gene associated with CD8^+^ T cell mediated immune responses (e.g. “T cell mediated cytotoxicity”). Furthermore, one of the downregulated genes was Mothers against decapentaplegic homolog 2 (SMAD2), a signal transducer that is activated by TGFβand plays and important role in apoptosis. The other up- and downregulated genes were associated with general functions such as metabolism, biosynthesis, transcription, ion transport and ion binding (Table 1 and Fig 4A). In addition, two of the most strongly downregulated genes (AIMP2/JTV1 and CXCR4) identified when comparing GagCM9^+^cells_SIV+RMs_ vs. CD8^+^cells_SIV-RMs_ also play roles in apoptosis. The other up and downregulated genes in this comparison were associated with general functions (e.g. protein transport, DNA binding, metabolism, cell signaling and ion binding) (Table 2 and Fig 4B). Finally, when comparing CD8^+^cells_SIV+RMs_ vs. CD8^+^cells_SIV-RMs_, CD8A was upregulated and AIMP2/JTV1 was downregulated (apoptosis-associated gene also downregulated between GagCM9^+^cells_SIV+RMs_ vs. CD8^+^cells_SIV-RMs_). The other up and downregulated genes were associated with functions such as protein transport, transcription, cell signaling and actin binding) (Table 3).

**Table 1:** The top 10 differentially over/under-expressed genes between GagCM9^+^cells_SIV+RMs_ vs. CD8^+^cells_SIV+RMs._

Gene name	Log_2_FC	Biological process or molecular function
Protein phosphatase 1 regulatory subunit 3F (PPP1R3F)	1.14	Glycogen metabolism
Discoidin domain-containing receptor 2 (DDR2)	1.01	Tyrosine kinase /ATP binding
Arginine—tRNA ligase, cytoplasmic (RARS)	0.96	Protein biosynthesis
Dipeptidyl peptidase 1 (CTSC)	0.95	T cell mediated Cytotoxicity
Protein phosphatase PTC7 homolog (PPTC7)	0.76	Metal ion binding
Actin-binding LIM protein 3 (ABLIM3)	-0.72	Zink ion binding
Platelet endothelial cell adhesion molecule (PECAM1)	-0.74	Cell adhesion
ATP synthase F(0) complex subunit C2, mitochondria (ATP5G2)	-0.77	Ion transport
Mothers against decapentaplegic homolog 2 (SMAD2)	-0.90	Signal transducer/Transcriptional modulator
Ectonucleoside triphosphate diphosphohydrolase 4 (ENTPD4)	-1.07	Hydrolase activity

**Table 2:** The top 10 differentially expressed genes between GagCM9^+^cells_SIV+RMs_ vs. CD8^+^cells_SIV-RMs_.

Gene name	Log_2_FC	Biological process or molecular function
Cyclin-dependent kinases regulatory subunit 2 (CKS2)	1.20	Cell cycle/Cell division
AP-2 complex subunit sigma (AP2S1)	1.20	Protein transport
Steroidogenic factor 1 (SF1)	1.19	DNA binding/ Transcriptional activator
GDP-fucose protein O-fucosyltransferase 2 (POFUT2)	1.18	Carbohydrate Metabolism
Discoidin domain-containing receptor 2 (DDR2)	1.14	Tyrosine kinase/Cell differentiation
RAC-gamma serine/threonine-protein kinase (AKT3)	-1.21	Protein serine/threonine kinase activity
60 kDa SS-A/Ro ribonucleoprotein (TROVE2)	-1.28	Metal ion binding
C-X-C chemokine receptor type 4 (CXCR4)	-1.37	Activation of MAPK activity/Apoptosis
Aminoacyl tRNA synthase complex-interacting multifunctional protein 2 (AIMP2/JTV1)	-1.58	Apoptosis
Testis-specific serine/threonine-protein kinase 3 (TSSK3)	-1.87	ATP binding/protein phosphorylation

**Table 3:** The top 10 differentially expressed genes between CD8^+^cells_SIV+RMs_ vs. CD8^+^cells_SIV-RMs._

Gene name	Log_2_FC	Biological process or molecular function
AP-2 complex subunit sigma (AP2S1)	1.475	Endocytosis/Protein transport
Polymerase I and transcript release factor (PTRF)	1.389	Transcription/Transcription termination
T-cell surface glycoprotein CD8 alpha chain (CD8A)	1.22	Adaptive immunity
Vacuolar protein sorting-associated protein 37C (VPS37C)	1.08	Protein transport
Actin-like protein 6A (ACTL6A)	1.05	Chromatin Binding
AP-1 complex subunit sigma-2 (AP1S2)	-1.06	Protein Transport
Actin-binding LIM protein 1 (ABLIM1)	-1.15	Actin binding
Aminoacyl tRNA synthase complex-interacting multifunctional protein 2 (AIMP2/JTV1)	-1.35	Apoptosis
Beta-2 adrenergic receptor (ADRB2)	-1.36	Activation of adenylate cyclase activity
Testis-specific serine/threonine-protein kinase 3 (TSSK3)	-1.88	ATP binding/protein phosphorylation

## Discussion

We have previously reported that the Gag CM9 Qdot 655 multimers can be used to detect SIV GagCM9^+^ cells in cryopreserved tissue and that the frequency of these cells, as detected by imaging analysis, was similar as the frequency detected by flow cytometry of single cell suspensions [27]. In this study we have demonstrated, for the first time to our knowledge, that it is possible to detect and laser-capture microdissect GagCM9^+^cells_SIV+RMs_, CD8^+^cells_SIV+RMs_ and CD8^+^cells_SIV-RMs_ from intact cryopreserved lymph node tissue sections of SIV infected and uninfected RMs with the use of these Gag CM9 Qdot 655 multimers and anti-CD8 antibodies. Additionally, the RNA obtained from the laser-capture microdissected CD8^+^ T cell populations could further be used to characterize their gene transcriptional profile via gene expression array analysis.

The GagCM9^+^cells_SIV+RMs_ and the CD8^+^cells_SIV+RMs_ displayed a relative even distribution of overexpressed and underexpressed genes as compared to CD8^+^cells_SIV-RMs_. Furthermore, the two CD8^+^ T cell populations within the SIV infected RMs were more similar to each other as compared to the CD8^+^ T cell populations from SIV infected vs. uninfected RMs. Thus, the lowest numbers of differentially expressed genes were detected in the comparison between GagCM9^+^cells_SIV+RMs_ vs. CD8^+^cells_SIV+RMs_, while GagCM9^+^cells_SIV+RMs_ vs. CD8^+^cells_SIV-RMs_ had the highest numbers of differentially expressed genes followed by the comparison between CD8^+^ cells_SIV+RMs_ vs. CD8^+^cells_SIV-RM_. Hierarchical clustering and heat maps were next used to visualize the differences in transcriptional profiles of these three enriched CD8^+^ T cell populations. The differentially expressed genes from GagCM9^+^cells_SIV+RMs_ vs. genes from CD8^+^cells_SIV+RMs_ were clearly distinguished and revealed that approximately half of the differentially expressed genes were upregulated and the other half was down regulated. Furthermore, the heat maps of GagCM9^+^cells_SIV+RMs_ vs. CD8^+^cells_SIV-RMs_ and CD8^+^cells_SIV+RMs_ vs. CD8^+^cells_SIV-RMs_ showed a similar gene expression pattern; again indicating the relatively even distribution of overexpressed and underexpressed genes between GagCM9^+^cells_SIV+RMs_ and the CD8^+^cells_SIV+RMs_ relative to CD8^+^cells_SIV-RMs._ Taken together, our data show that the methods used allow us to discriminate between the three different CD8^+^ T cell populations which display both distinct and unique genes. Thus, the transcriptional profile of SIV Gag specific CD8^+^ T cells (GagCM9^+^cells_SIV+RMs_) was clearly distinguished from the other CD8^+^ T cells residing in lymph nodes of SIV infected RMs (e.g. CD8^+^cells_SIV+RMs_). However, since these CD8^+^ T cells were only selected based on their expression of CD8, we cannot rule out that some of the CD8^+^cells_SIV+RMs_ may also be SIV specific CD8^+^ T cells restricted by other than the Mamu-A*01 allele. The RMs included in this study were only genotyped for Mamu-A*01, although RMs obviously can express a variety of Mamu-A and -B alleles [33, 34]. Furthermore, our study was not powered for the purpose of differential expression analysis and thus we cannot be certain on the functional relevance of these gene sets. Nevertheless, our data is in line with a recent publication by Pereyra *et al* that stresses the importance of being able to discriminate between the specific protective and non-protective HIV specific CD8^+^ T cells epitopes in order to specify which epitopes should be included in a HIV vaccine [13]. Thus further studies of detailed evaluations of immunodominant versus subdominant epitopes are warranted and may help provide insights on the *in vivo* mechanisms of SIV CD8^+^ T-cell interactions [2, 13].

Two genes of particular interest, when comparing GagCM9^+^cells_SIV+RMs_ vs. CD8^+^cells_SIV+RMs_, were CTSC (upregulated) and SMAD2 (downregulated). The CTSC gene encodes the protein Cathepsin C, which is a key enzyme in the activation of granule serine peptidases, including Granzyme A and B which play a major role in cytotoxic CD8^+^ T cell mediated killing [35]. The signal transducer SMAD2 is activated by TGFβ and this signaling pathway is suggested to play an important role in apoptosis as well as in virus specific CD8^+^ T cell responses during chronic viral infections, such as LCMV infection in mice and HIV/SIV infection in humans and NHP, respectively [36–38]. Sustained activation of the SMAD/TGFβ signaling pathway in mouse models may however result in suppression of viral specific CD8^+^ T cells and hence viral persistence [38] and attenuation or blocking of this pathway can restore viral specific T cell responses, including those targeting HIV/SIV [36–38]. It is interesting to speculate if these differences in SMAD/TGFβ signaling plays a role in potential functional differences of GagCM9 specific CD8^+^ T cells from other CD8^+^ T cells present in lymph nodes of SIV infected RMs. In addition, when comparing GagCM9^+^cells_SIV+RMs_ vs. CD8^+^cells_SIV-RMs_ and CD8^+^cells_SIV+RMs_ vs. CD8^+^cells_SIV-RMs_, two downregulated genes (AIMP2/JTV1 and CXCR4) involved in apoptosis were identified. Thus, the observed downregulation of genes involved in apoptosis in all three comparisons raises the question whether GagCM9^+^cells_SIV+RMs_ are less prone to become apoptotic.

Our studies were designed to be exploratory and hence more provocative in generating hypotheses than definitive in their conclusions. Our sample size was small, which limited statistical power and precluded us from incorporating multiple comparison correction techniques for doing comparisons of gene transcription data. In this exploratory study, we used the human Illumina bead array since less amount of cDNA was required as compared to the NHP Illumina bead array. Furthermore, the SIV infected RMs are Mamu-A*01 positive while the SIV uninfected monkey is Mamu-A*01 negative. RMs expressing Mamu-A*01 are associated with a slow SIV disease progression and the SIV specific GagCM9 CD8^+^ T cells have been suggested to play an important role in this slow disease progression [9]. However, the slow disease progression seen in Mamu-A*01 may also be due to the co-expression of Mamu-B*17 or–B*08 that are highly associated with elite control of SIV [33, 34]. Thus, it would have been preferable if the RMs used in this study were genotyped for additional MHC class I alleles such as Mamu-A*02, -B*17 or–B*08, which presents several immunodominant epitopes [33, 34]. While keeping these caveats in mind, our data imply that the three different CD8^+^ T cell populations assessed here displayed distinct gene transcriptional profiles. Interestingly, genes involved in cytotoxic killing, and apoptosis were altered in the GagCM9^+^ cells_SIV+RMs_ as compared to the other CD8^+^ T cells from SIV infected and from uninfected RMs. Thus it would be interesting to further investigate the role of these genes in SIV specific CD8^+^ T cells as compared to other subdominant CD8^+^ T cells. Furthermore, GagCM9^+^cells_SIV+RMs_ and CD8^+^cells_SIV+RMs_ showed a rather similar gene expression profiles as compared to CD8^+^ T cells of uninfected RMs suggesting that SIV is a major driver of gene transcription in the CD8^+^ T cell mediated immune responses in SIV infection.

Several studies have shown transcriptional differences in immune cells obtained from HIV infected individuals in different clinical stages of the disease, as well as from SIV infected vs. uninfected, pathogenic vs. non-pathogenic and vaccinated vs. unvaccinated NHPs [26, 39–54]. In a majority of these studies the transcriptional profiles of PBMCs or of single cell suspensions of lymph nodes were investigated, however some of the studies also examined the transcriptional profile of purified CD8^+^ T cells from blood [51–54]. The major contribution of our study is the implementation of methods allowing detection, collection and capturing of specific cells of interest from intact tissue sections and subsequent analyses of the different enriched cell populations. Hence, the data provided reflects the status of the cells within their “natural environment” at time of sampling and at critical sites of immune reactions.

In summary, we here introduced novel techniques that allows discrimination between three different CD8^+^ T cell populations, namely GagCM9 specific CD8^+^ T cells and other CD8^+^ T cells from intact cryo preserved lymphoid tissues of SIV infected RMs as well as CD8^+^ T cells from uninfected RMs. These different CD8^+^ T cell populations displayed unique transcriptional profiles. In order to specify which epitopes should be included in a HIV vaccine is of major importance to be able to distinguish between the specific protective and non-protective HIV specific CD8^+^ T cells epitopes. Single-cell gene expression analysis of antigen specific T cells furthermore suggest that there is a great heterogeneity between individual cells, even with a well-defined cell population, which may be missed when averaging gene expression within a given cell population [55–57]. Thus, the combination of the *in situ* staining and LCM techniques used in this study could be a potential methodological platform in combination with single-cell gene or protein expression analysis, for further characterization of CD8^+^ T cells of importance for the control of SIV/HIV. Consequently, a larger sample size, which also takes into consideration the effect of different MHC class I alleles, is thus needed to pinpoint the gene transcriptional profiles of SIV specific CD8^+^ T cells, particular in lymphoid tissue where the main HIV/SIV replication occurs.

## Supporting Information

S1 Table Differentially expressed genes between GagCM9+cellsSIV+RMs vs. CD8+cellsSIV+RMs. (XLS)

S2 Table Differentially expressed genes between GagCM9+cellsSIV+RMs vs. CD8+cellsSIV-RMs. (XLS)

S3 Table Differentially expressed genes between CD8+cellsSIV+RMs vs. CD8+cellsSIV-RMs. (XLS)
